# Creating an Innovative Artificial Intelligence–Based Technology (TCRact) for Designing and Optimizing T Cell Receptors for Use in Cancer Immunotherapies: Protocol for an Observational Trial

**DOI:** 10.2196/45872

**Published:** 2023-07-13

**Authors:** Joanna Bujak, Stanisław Kłęk, Martyna Balawejder, Aleksandra Kociniak, Kinga Wilkus, Rafał Szatanek, Zofia Orzeszko, Joanna Welanyk, Grzegorz Torbicz, Mateusz Jęckowski, Tomasz Kucharczyk, Łukasz Wohadlo, Maciej Borys, Honorata Stadnik, Michał Wysocki, Magdalena Kayser, Marta Ewa Słomka, Anna Kosmowska, Karolina Horbacka, Tomasz Gach, Beata Markowska, Tomasz Kowalczyk, Jacek Karoń, Marek Karczewski, Mirosław Szura, Anna Sanecka-Duin, Agnieszka Blum

**Affiliations:** 1 Ardigen SA Cracow Poland; 2 Department of Physics and Biophysics Institute of Biology Warsaw University of Life Sciences Warszawa Poland; 3 Surgical Oncology Clinic Maria Sklodowska-Curie National Research Institute of Oncology Cracow Poland; 4 Department of General and Oncological Surgery Brothers Hospitallers Hospital Cracow Poland; 5 Department of General Surgery and Surgical Oncology Ludwik Rydygier Memorial Hospital Cracow Poland; 6 Colon Cancer Unit Department of Oncological Surgery Voivodeship Multi-Specialist Center for Oncology and Traumatology Lodz Poland; 7 Holy Cross Cancer Center Clinic of Clinical Oncology Cracow Poland; 8 Department of General Surgery Andrzej Frycz Modrzewski Krakow University Cracow Poland; 9 Department of General and Transplant Surgery Poznan University of Medical Sciences University Hospital Poznan Poland; 10 General and Colorectal Surgery Department J Struś Multispecialist Municipal Hospital Poznan Poland; 11 Surgical Clinic Institute of Physiotherapy Faculty of Health Sciences Jagiellonian University Medical College Cracow Poland

**Keywords:** AI, artificial intelligence, colorectal cancer, HLA, human leukocyte antigen, immunotherapy, neoantigen, T cell receptors, TCR

## Abstract

**Background:**

Cancer continues to be the leading cause of mortality in high-income countries, necessitating the development of more precise and effective treatment modalities. Immunotherapy, specifically adoptive cell transfer of T cell receptor (TCR)-engineered T cells (TCR-T therapy), has shown promise in engaging the immune system for cancer treatment. One of the biggest challenges in the development of TCR-T therapies is the proper prediction of the pairing between TCRs and peptide-human leukocyte antigen (pHLAs). Modern computational immunology, using artificial intelligence (AI)-based platforms, provides the means to optimize the speed and accuracy of TCR screening and discovery.

**Objective:**

This study proposes an observational clinical trial protocol to collect patient samples and generate a database of pHLA:TCR sequences to aid the development of an AI-based platform for efficient selection of specific TCRs.

**Methods:**

The multicenter observational study, involving 8 participating hospitals, aims to enroll patients diagnosed with stage II, III, or IV colorectal cancer adenocarcinoma.

**Results:**

Patient recruitment has recently been completed, with 100 participants enrolled. Primary tumor tissue and peripheral blood samples have been obtained, and peripheral blood mononuclear cells have been isolated and cryopreserved. Nucleic acid extraction (DNA and RNA) has been performed in 86 cases. Additionally, 57 samples underwent whole exome sequencing to determine the presence of somatic mutations and RNA sequencing for gene expression profiling.

**Conclusions:**

The results of this study may have a significant impact on the treatment of patients with colorectal cancer. The comprehensive database of pHLA:TCR sequences generated through this observational clinical trial will facilitate the development of the AI-based platform for TCR selection. The results obtained thus far demonstrate successful patient recruitment and sample collection, laying the foundation for further analysis and the development of an innovative tool to expedite and enhance TCR selection for precision cancer treatments.

**Trial Registration:**

ClinicalTrials.gov NCT04994093; https://clinicaltrials.gov/ct2/show/NCT04994093

**International Registered Report Identifier (IRRID):**

DERR1-10.2196/45872

## Introduction

### Overview

Cancer has been recognized as a leading cause of death in high-income countries [1]. Currently, cancer treatment is based on 1 of the 3 available modalities: surgery, radiotherapy, and/or chemotherapy. However, recent research advancements in the field of cancer molecular biology have contributed to the development of novel tumor treatment approaches, such as targeted therapies and immunotherapies [2].

Immunotherapy uses the immune system against cancer cells by using monoclonal antibodies, tumor vaccines, or adoptive cell transfer (ACT). In 2013, immunotherapy was announced as the breakthrough technology of the year by the prestigious journal *Science*, while in 2018, two immunologists, James P Allison and Tasuku Honjo, were awarded the Nobel Prize in Medicine and Physiology for the discovery of the so-called immune checkpoints: programmed death receptor 1 and cytotoxic T cell antigen 4 [3,4]. They have shown that antibodies blocking these receptors, known as immune checkpoint inhibitors, cause reactivation of T cells, thus allowing them to attack and eradicate cancer cells. The first immune checkpoint inhibitor (ipilimumab) was approved by the US Food and Drug Administration (FDA) in 2011 to treat metastatic melanoma [5]. Recognition of immunotherapy as a cancer treatment modality paved the way for further studies and the development of other immunotherapeutic strategies.

### Advancements in ACT for Cancer Treatment

ACT is a type of immunotherapy where tumor-reactive immunocytes are infused into a patient with cancer to eradicate tumor cells and maintain durable immune responses. To date, T lymphocytes are the most commonly used immune cells in ACT. T cells are responsible for a specific recognition of tumor neoantigens and subsequent destruction of malignant cells. The idea that tumor-reactive T cells can be used for cancer treatment evolved from early studies in 1991 by van der Bruggen et al [6] who showed that melanoma cells express antigens presented by the human leukocyte antigen (HLA)-A1 molecule and are specifically recognized by cytotoxic T cells. Importantly, the authors underlined that the presented antigen was not found in the panel of healthy tissues, which raised the possibility of the development of a targeted immunotherapy for patients with melanoma. Further studies and the subsequent discovery of tumor antigens, such as melanoma antigen recognized by T-cells 1 (MART-1), glycoprotein 100, or New York esophageal squamous cell carcinoma 1 (NY-ESO-1), proved that T cells are able to specifically recognize tumor antigens [7].

Initially, ACT treatment was based on tumor-infiltrating lymphocytes (TILs) that were isolated from the tumor lesion and expanded in vitro. However, one of the major limitations of this approach was associated with the fact that it only applied to resectable tumors and to tumors that were infiltrated by T cells. Furthermore, ex vivo expansion of TILs was proven to be problematic and time-consuming [8,9]. To bypass these limitations, an approach using blood-derived T cells, engineered ex vivo to recognize tumor antigens and then infused back into the patient, was introduced as an alternative.

### Chimeric Antigen Receptor and T Cell Receptor T Cells

Currently, there are 2 main approaches to retarget T cells: chimeric antigen receptor (CAR)- and T cell receptor (TCR)-based. Despite the fact that both approaches involve redirecting T cell specificity, their mechanisms of action are different.

CAR-T cells use a single-chain fragment variable from monoclonal antibodies to specifically recognize tumor antigens that are expressed on the cell surface [10]. Spectacular results were achieved when CAR-T cells were used against the cluster of differentiation (CD) 19 molecule, leading to a complete remission in many patients with B-cell acute lymphoblastic lymphoma (B-ALL). This resulted in the first FDA-approved genetically engineered cell therapy in 2017 [11].

On the other hand, TCR-based technology uses heterodimers of α and β chains that recognize peptides in the context of HLA. The major advantage of this technology over CAR-T cells is that TCRs recognize intracellular antigens that are presented by major histocompatibility complex molecules [10,12]. In 2006, Morgan et al [13] demonstrated that ACT treatment using autologous TCR-engineered T cells (TCR-T) to specifically recognize the MART-1 antigen mediated a durable regression of metastatic melanoma lesions. Similarly, phase I and II clinical trials with TCR-engineered autologous lymphocytes reprogramed to express high-affinity TCR against NY-ESO-1 and L-antigen 1, cancer-testis antigens, showed clinical response in 80% of patients with advanced myeloma with 70% demonstrating a complete response to the treatment [14]. These results not only showed that autologous T cells can be redirected by introducing TCR with defined specificity and affinity against tumor antigens, but also provided hope for the treatment of incurable metastatic disease. Despite the promising results, TCR-based technology faces several drawbacks associated with (1) finding a good antigenic peptide presented by HLA, (2) screening for a TCR that binds a given peptide-human leukocyte antigen (pHLA), (3) optimizing affinity and avidity of the pHLA:TCR complex, and (4) performing safety evaluations, all of which require extensive laboratory work. This is why a search for artificial intelligence (AI) models to address these issues has been gaining substantial interest in the past few years.

### TCR-T Therapy With a pHLA:TCR Database

The use of AI in medicine has been gaining momentum in recent years. There have been successful attempts at applying AI in clinical histopathology [15,16], mammogram analysis [17], and immuno-oncological studies involving checkpoint inhibitors [18]. Furthermore, recent studies have shown that deep learning can be successfully applied for the prediction of TCR-peptide binding specificity [19-21] at least for some of the possible validation scenarios (eg, the scenarios single peptide binding, TCR peptide pairing-I to TCR peptide pairing-III [20]), with the general problem (predictions for pHLA complexes and TCRs unseen by the AI models during training) being still challenging for the current methods [21].

In TCR-T cell therapies, a reliable approach for predicting pHLA:TCR binding is of great importance although it presents challenges, which should be properly addressed. One of the major obstacles concerns the data available in the public domain to train such models, which is often limited or incomplete. In many cases, these data describe only 1 chain, mainly the β chain of TCR, while data on the α chain are missing. This is a major drawback to screening for antigen-binding TCRs, since the complementarity determining region 3 loops sequences of both α and β chains determine the TCR binding specificity [20]. It is important to emphasize that currently, there are no available solutions that allow for in silico predictions of TCR sequences binding a specific, novel peptide in the context of HLA. The algorithms that are used for TCR sequencing data analysis do not provide data regarding matched pHLA complexes. Similarly, bioinformatics tools for the prediction of TCR antigen binding for human TCRs offer results for a limited number of known epitopes. Additionally, the evaluation of possible toxicities associated with TCRs requires extensive and expansive in vitro experiments.

**Figure 1:**
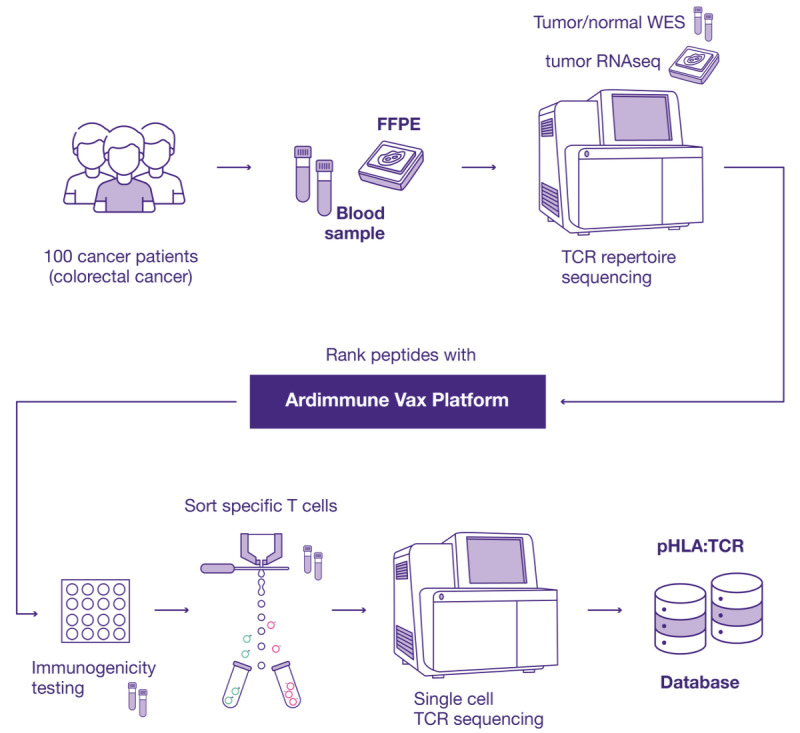
Schematic overview of the workflow required for the generation of the proposed pHLA:TCR (peptide-human leukocyte antigen: T cell receptor) database. FFPE: formalin-fixed paraffin-embedded; RNAseq: RNA sequencing; WES: whole exome sequencing.

Herein, we present the study protocol for collecting patient material necessary to build a pHLA:TCR database for training and validating AI technology that can be used for screening and optimizing TCRs for TCR-T cell therapies (Figure 1). As highlighted above, the current lack of proper data training for AI-based pHLA:TCR interaction prediction models poses a major limitation in the discovery of specific TCRs. Therefore, in our opinion, creating a comprehensive database is the first and most fundamental step in building an efficient AI model for TCR discovery. We believe that the incorporation of AI technology will not only accelerate and advance the entire process relating to the identification of specific TCRs but will also provide a new approach to personalized cancer treatment using therapeutic T cells.

## Methods

### Study Design and Objectives

This is a multicenter observational study performed by the surgical oncology departments in Poland: the Maria Sklodowska-Curie National Research Institute of Oncology in Cracow, Brothers Hospitallers of Saint John of God Hospital in Cracow, Ludwik Rydygier Memorial Hospital in Cracow, St Raphael Hospital in Cracow, Voivodeship Multi-Specialist Center for Oncology and Traumatology in Lodz, Holy Cross Cancer Center in Kielce, J Struś Multispecialist Municipal Hospital, and Heliodor Swiecicki Clinical Hospital in Poznan. The overall objective of this study is to create a pHLA:TCR database to aid the development of an AI-based platform for the selection of specific TCRs used in cancer immunotherapy. This aim will be achieved by collecting high-quality blood and tissue samples from patients with colorectal cancer (CRC) that can be used for next generation sequencing, single-cell, and bulk TCR repertoire sequencing. The sequencing data will be processed with Ardigen (Ardigen SA)-developed platforms to generate a pHLA:TCR database.

### Ethical Considerations and Regulatory Approvals

This study has been approved by the Maria Sklodowska-Curie National Research Institute of Oncology, Gliwice branch (KB/430-87/21). It was carried out in accordance with the Declaration of Helsinki and the Good Clinical Practice guidelines issued by the International Conference on Harmonization (ICH-E6, 17/07/1996). The enrolled participants were evaluated based on the inclusion and exclusion criteria. The selected participants provided written informed consent before the start of all experimental procedures. All the participants were provided with a thorough explanation of the proceedings planned in the study with an emphasis on the type and quantity of biological samples that will be collected from them. Furthermore, the participants were informed on the type of experiments that will be performed and how the samples will be stored. Generated data reports will be confidential and stored safely at a repository at Ardigen.

### Patient Selection

All the patients with CRC admitted to the surgical oncology wards of the participating hospitals were evaluated for their study eligibility. All the participants were required to be older than 18 years with a diagnosed and histopathologically confirmed advanced resectional CRC adenocarcinoma in active stage II, III, or IV. Participants were excluded from the study if they were unwilling to sign the informed consent form; had a history of inflammatory bowel disease, mucinous adenocarcinoma of the colon, cancer of the anal canal and verge, or adenocarcinoma of the appendix; had a histopathological diagnosis other than adenocarcinoma; were undergoing neoadjuvant and radiotherapies; were pregnant; had HIV infection or active hepatitis B or C infections; or had a leukocyte (white blood cell) levels <4000/mm^3^. Qualified participants agreed to make the formalin-fixed paraffin-embedded (FFPE) tissue from the primary tumor available for research as well as to have their blood drawn.

### Study Procedure

After the selection process, the enrolled participants underwent medical history review and physical examination by a medical doctor of the research team. Once completed, tissue samples of the primary tumor and blood were obtained from the participants. The tissue samples were provided in the form of FFPE blocks with a total area of embedded tissue of at least 100 mm^2^ or alternatively as a minimum of 10 histopathological slides, the quality of which was confirmed by a pathologist. Blood sample collection was performed twice by qualified medical personnel (ie, a nurse or medical doctor) after which the sample was secured for further processing.

Study participants had the option of terminating their participation in the study at any given time. The participants could have also been withdrawn from the study at the discretion of the investigational team.

### Sample Processing

#### Sample Collection

Biological samples that were obtained from the study participants included FFPE blocks of the primary tumor tissue or histopathological slides derived from it and their peripheral blood samples. The FFPE blocks and histopathological slides were provided by the hospital laboratory or department of pathomorphology of the participating institution. Peripheral blood samples were collected into heparin-containing tubes in 2 blood draws, 40 mL each. Additionally, at the first venipuncture, 2.5 mL of peripheral blood was collected per 1 PAXgene Blood DNA and 1 PAXgene Blood RNA tube (Thermofisher) for DNA and RNA isolation, respectively. If an insufficient number of peripheral blood mononuclear cells (PBMCs; <50 million) was obtained after the first 2 draws, an additional 40 mL of blood was collected from eligible participants (eg, those who had not yet started chemotherapy) after receiving their consent. Samples were transported to the Biobank Lab at the Head Department of Oncobiology and Epigenetics, Faculty of Biology and Environmental Protection, University of Lodz, according to the standard operating procedures. For long-term storage, the samples in PAXgene Blood DNA and PAXgene Blood RNA tubes are stored at –80 °C, and FFPE blocks and histopathological slides are stored at 2-8 °C.

#### PBMC Isolation

Blood collected into heparin-containing tubes was used for PBMC isolation. The isolation procedure for all samples started within 3 hours after venipuncture and was performed under sterile conditions according to standard density gradient centrifugation protocol. Briefly, blood samples were diluted with a phosphate-buffered solution in a 1:1 ratio at room temperature (RT) and layered onto Histopaque-1077 reagent (Sigma-Aldrich) in 50 mL test tubes. Next, the samples were centrifuged according to the manufacturer’s instructions (at 400*g* at RT for 30 minutes without braking or acceleration). After centrifugation, the leukocyte phase was transferred into new tubes and washed with phosphate-buffered saline followed by centrifugation at 300*g* at RT for 10 minutes. To remove erythrocytes, the cell pellet was treated with a red blood cell lysis buffer according to the manufacturer’s instructions. The viability of the isolated PBMCs was assessed using trypan blue staining. If applicable, the PBMCs were resuspended in fetal bovine serum with 10% of dimethyl sulfoxide and cryopreserved in liquid nitrogen for long-term storage.

#### DNA and RNA Isolation

DNA and RNA were isolated from tumor FFPE block samples after macrodissection and evaluation of corresponding hematoxylin and eosin (H&E)-stained slides with tumor area distinctly labeled by a pathomorphologist.

For DNA and RNA extraction from blood samples collected in the PAXgene Blood DNA and PAXgene Blood RNA tubes, the PAXgene Blood DNA and PAXgene Blood RNA kits (Qiagen) were used, respectively, according to the manufacturer’s protocol.

#### Next Generation Sequencing

Isolated DNA from both FFPE blocks and peripheral blood was prepared for whole exome sequencing (WES) sequencing with commercial kits for 100 bp paired-end read lengths. The RNA isolated from FFPE blocks was used for RNA library preparation for paired-end sequencing with read lengths of 100 bp. The quality of prepared libraries was checked with Fragment Analyzer and sequenced on an Illumina sequencer with expected depth for DNA of 150x and 300x for PBMC and FFPE samples, respectively. RNA sequencing was set to obtain at least 100 million reads for each sample.

### Identification of Immunogenic Peptides and Sequencing TCRs That Bind Them

ARDesign (previously called ArdimmuneVax) is an Ardigen-developed platform that uses in-house created AI models to design off-the-shelf and personalized peptide-based cancer vaccines and target identification for TCR-based therapies [22,23]. ARDesign platform includes ARDitox, the ARDisplay—presentation model, Ardimmune—immunogenicity model, and Meta-model, which considers the output of these models and other metrics. DNA sequences will be aligned to the reference genome and both somatic and germline mutation will be called, which will constitute one of the inputs for the ARDesign platform. Expression profiles derived from RNA sequences will comprise the other input for the platform.

Next, based on ARDesign results, peptide immunogenicity can be validated by in vitro functional assays (ie, ELISpot), and fluorescently labeled pHLA multimers can be synthesized and applied for the isolation of reactive T cells with a flow cytometry cell sorting methodology. For that purpose, the isolated, biobanked PBMCs will be stained with fluorescent pHLA multimers and a panel of antibodies allowing the gating of pHLA-specific CD8^+^ T cells.

Sorted CD8^+^ T cells will be analyzed using the 10x Genomics (10x Genomics, Inc) single-cell technology to obtain paired α and β sequences of the TCRs with information about the pHLA that they bind.

Additionally, RNA isolated from the peripheral blood collected into PAXgene Blood RNA tubes will be used for bulk TCR repertoire sequencing to enable profiling of TCR repertoire diversity and clonality and to help to prepare negative training data sets for the pHLA:TCR database.

**Figure 2:**
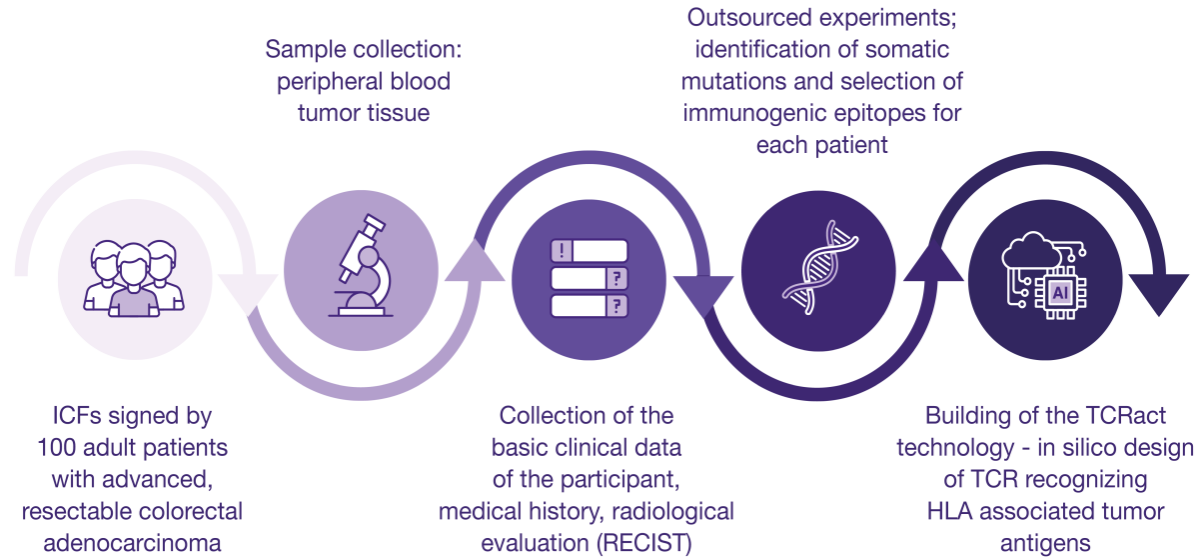
Overview of creating an artificial intelligence–based technology (TCRact) for T cell receptors (TCRs) design and optimization. HLA: human leukocyte antigen; ICF: informed consent form; RECIST: response evaluation criteria for solid tumors.

Altogether, the gathered results can serve as the basis to generate a reliable pHLA:TCR database that includes validated TCRαβ-paired sequences of a known peptide specificity. The overall process of the proposed pHLA:TCR database generation is presented in Figure 2.

## Results

Participant recruitment and sample collection started in October 2021 and was completed at the end of 2022. To date, primary tumor (FFPE blocks) and peripheral blood samples have been obtained from 100 participants. Isolation of PBMCs from peripheral blood has been performed for all 100 patients. On average, 74 million PBMCs were isolated from 80 mL of blood with average cell viability of 93%. All the isolated PBMCs were cryopreserved and are being stored at the Biobank Lab, University of Lodz.

**Figure 3:**
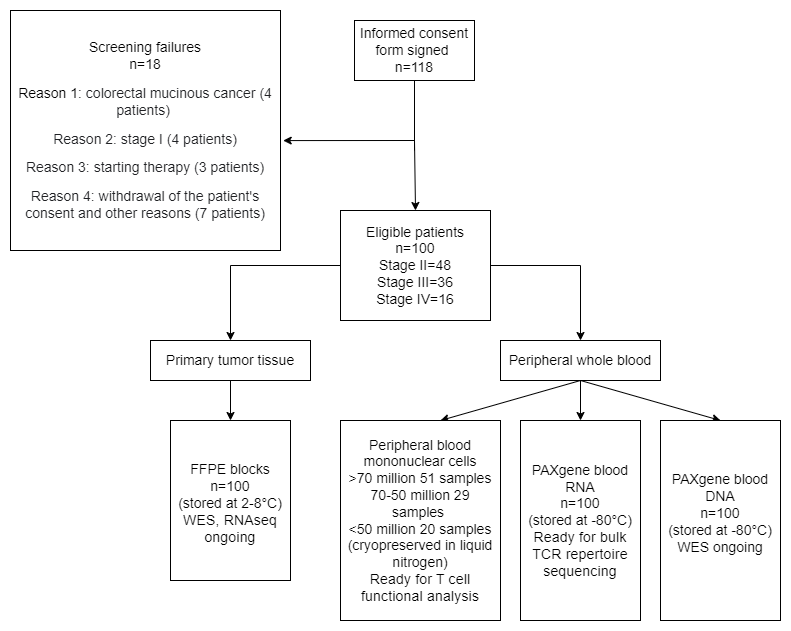
Flowchart of participants, specimens, and samples through the study. FFPE: formalin-fixed paraffin-embedded; RNAseq: RNA sequencing; TCR: T cell receptor; WES: whole exome sequencing.

To date, 85 participants have had their DNA and RNA extracted from both FFPE and peripheral blood samples. In 57 cases, WES was performed to analyze the occurrence of somatic mutations. Altered gene expression analysis was done for 57 participants using the RNA sequencing method. Additionally, RNA samples (isolated from blood) will be undergoing bulk TCR repertoire sequencing to enable profiling of TCR repertoire diversity and clonality. In all of the cases mentioned above, the obtained sequencing data (DNA and RNA) were incorporated into the ARDesign platform to rank the immunogenic potential of tumor peptides. Final study results are expected to be analyzed by September 2023. For further information regarding the progress of the study refer to the study flowchart presented in Figure 3.

## Discussion

### Overview

This study describes a protocol of a trial for creating a comprehensive database containing pHLA-TCR sequences with paired α and β chains of TCRs. The generated database will be used for the development and testing of an AI-based platform designed for the prediction and optimization of TCR sequences that specifically recognize tumor antigens. These types of platforms are of great interest, especially for the development of antitumor immunotherapies using TCR-T cells. ACT trials using TCR-T cells or CAR-T cells have shown that complete and durable eradication of tumor cells in the primary tumor is possible [24]; however, there are also reports suggesting that this type of immunotherapy might be effective in the treatment of metastatic disease [25,26], which is a major challenge for current treatment modalities. A great example of ACT efficacy against neoplastic disease is a therapy involving CD19 CAR-T cells in the treatment of relapsed or refractory B-ALL and relapsed or refractory diffuse large B-cell lymphoma in adult patients. This therapy was approved by FDA in 2017, and in 2018 it was authorized by the European Medicines Agency, which underlines that these technologies are highly promising for the treatment of neoplastic diseases [27]. Nevertheless, despite the spectacular effect of CAR-T cells in the treatment of some blood cancers, these types of cells are not as effective in the treatment of solid tumors [28]. TCR-T cell therapy appears to be a better approach for the treatment of solid tumors [29], which make up the majority of all cancer cases. Furthermore, unlike CAR-T technology, TCR-T cells are able to recognize mutated intracellular proteins, which make up a much larger part of cellular proteins than surface antigens by themselves [30,31]. In other words, the range of potential antigen targets is larger in the case of TCRs than CARs. Thus, the development of TCR-T technology might offer hope for “universal” cancer treatment. For this reason, many pharma and biotech companies are involved in the development of TCR-based therapies and clinical trials involving patients with cancer. Nevertheless, the advancement of TCR-T technologies had been slowed down by several drawbacks. The main obstacle with TCR-engineered ACT is associated with finding the appropriate TCRs that specifically recognize tumor antigens and do not show “on-target, off-tumor” toxicities at the same time. Another challenge is finding a TCR with proper avidity for the target. Currently, the development of TCR-T cells for therapeutic purposes is based on extensive lab work, which is time-consuming, labor-intensive, and very expensive.

For this reason, our study aims to develop a platform for faster TCR selection and simultaneous prediction of potential toxicities. Our platform is intended not only to accelerate TCR identification but also to reduce the cost of the entire process. One of the main stages of TCRact platform development is to create a unique pHLA:TCR database. Currently, high-throughput TCR sequencing enables deep analysis of the TCR repertoire, which allows for comparison between different tissues and individuals. Currently, several databases of TCR sequences are available, such as VDJdb, McPAS-TCR, and IEDB, and subsequently, algorithms that allow for TCR specificity prediction have also been developed (ie, TCRdist and TCRex) [32-36]. It has to be stressed that the available databases are often incomplete and, in many cases, provide a sequence of only one of the TCR chains, or both α and β chains that are not paired. Furthermore, in many cases, they lack information about the cognate peptides and HLA types involved in peptide presentation. Hence, the function of only a minor fraction of TCRs is known, especially in the context of TCR-peptide-HLA interactions. Recently, Zvyagin et al [37] pointed out this problem by concluding that there is a lack of studies and protocols that allow for good prediction of TCRs and epitopes based on their sequences.

To build the database with a size that is comparable to the publicly available data set, we are aiming to use samples from approximately 100 patients [38]. Notably, our database will be based on the data obtained from the primary tumor and blood samples of patients with cancer. This approach is more specific (as tumor neoantigens are highly similar to self-antigens, unlike viral or bacterial antigens) for the identification of the TCR-peptide-HLA complex in the context of neoplastic disease in comparison to TCR data obtained from infectious diseases or vaccine monitoring studies [15,34,39,40]. Our approach helps to populate the database with a unique set of sequences that may be invaluable in the context of immuno-oncological therapies using TCR-T cells. For this reason, close cooperation with clinical sites guaranteeing the highest quality of the collected biological specimens together with complete clinical data is essential for the completion of this project’s goals.

In this study, an AI-based platform is being developed as a tool for designing a precise and safe immunotherapeutic approach in tumor treatment. Adopting this type of approach would allow oncogenic, personalized immunotherapy to be widely accessible to the general public.

### Limitations

Although we strongly believe that TCR-T therapies developed with the aid of AI-based platforms, such as the one discussed in this study, will become more accessible for cancer treatment with time, there are still a number of limitations that need to be addressed. The first major obstacle concerns the sharedness of the target (ie, neoantigen and tumor-specific antigen) for such therapies. There is a limited number of defined shared peptide targets. Furthermore, the method for creating the pHLA:TCR database described in this study will mainly provide data for personal neoantigens (specific to a given patient) and the TCRs that bind them. However, we believe that including neoantigens in the database might address the key limitation in pHLA:TCR binding prediction with AI models and the limited diversity of pHLAs in the training set. Another limitation to consider involves the number of patients in the study cohort itself and the number of pHLA:TCR pairs that it can potentially generate. Our cohort is composed of 100 patients, and we plan to choose 1 pHLA per patient. However, it is difficult to predict what number of different T cell clones per pHLA will be identified in our experimental approach, as the number can differ greatly depending on epitope sequence, clonal diversity, and clone size. In summary, the pHLA:TCR database that we plan to build using the approach described in this study may turn out to be of modest size. However, we believe that it will make a significant contribution to the field because our data set will provide information about specific cancer antigens that are currently underrepresented in public databases.

### Conclusions

The results of this study provide the basic information for the development of an innovative platform that allows for fast and safe in silico prediction of TCRs to be used in cancer immunotherapy.

## Abbreviations

ACT: adoptive cell transfer
AI: artificial intelligence
B-ALL: B-cell acute lymphoblastic lymphoma
CAR: chimeric antigen receptor
CD: cluster of differentiation
CRC: colorectal cancer
FDA: Food and Drug Administration
FFPE: formalin-fixed paraffin-embedded
H&E: hematoxylin and eosin
HLA: human leukocyte antigen
MART-1: melanoma antigen recognized by T-cells 1
NY-ESO-1: New York esophageal squamous cell carcinoma 1
PBMC: peripheral blood mononuclear cell
pHLA: peptide-human leukocyte antigen
RT: room temperature
TCR: T cell receptor
TCR-T: TCR-engineered T cell
TIL: tumor-infiltrating lymphocyte
WES: whole exome sequencing

## Data Availability

The data sets generated and analyzed in this study are available from the corresponding author on reasonable request.
